# Study of silanized nanostructures with immobilized fumarase for production of L-malate

**DOI:** 10.55730/1300-0527.3469

**Published:** 2022-07-19

**Authors:** Nevra ÖZTÜRK ATAY, Kevser KUŞAT, Sinan AKGÖL

**Affiliations:** 1 Department of Application and Research Center for Testing and Analysis, Ege University, İzmir, Turkey; 2 Department of Chemistry, Faculty of Science, Dokuz Eylül University, İzmir, Turkey; 3 Department of Biochemistry, Faculty of Science, Ege University, İzmir, Turkey

**Keywords:** Fumarase, nanostructure, L-malate production, immobilized enzyme

## Abstract

The conversion of fumaric acid into L-malate by fumarase immobilized on silanized nanostructures was analyzed experimentally. The enzyme was bound to the silanized nanostructures. We carried out scanning electron microscopy (SEM), fourier transform infrared spectroscopy (FTIR) analysis, zeta size analysis and surface area calculation for the characterization of the nanostructures. The effect of initial enzyme concentration and pH on immobilization procedure were investigated and the change of Michaelis-Menten constants (K_m_ and V_max_) with immobilization was examined. The change in the storage stability of the enzyme by immobilization was also investigated. The stability of the immobilized enzyme was very good. We observed that the fumarase was bound to silanized nanostructures [p(HEMA)-3-MTES] in much greater amounts. We have compared the activities of free fumarase and immobilized fumarase and we have observed a significant increase in the activity of the fumarase after immobilization for L-malate production. Moreover, we came to the conclusion that this activity can be better preserved for 30 days compared to free fumarase.

## 1. Introduction

Studies performed by immobilizing microbial cells on different materials are frequently included in the literature. Takata et al. [1] investigated fumarase activity by trapping Brevibacterium flavum cells into K-carrageenan. A similar study was conducted for the production of L-malic acid by Saccharomyces Cerevisiae trapped in polyacrylamide gel spheres by Oliveria et al. [2]. Noude and Nicol [3] performed a study on the production of L-malic acid using immobilized Rhizopus oryzae. The major disadvantage of such studies in the literature is the stability problems of microbial cells [1,4] and the possibility of by-product formation. It is also necessary to pay attention to the complex system of the enzyme-cell membrane-cell wall-gel matrix where immobilized microbial cells are used. It may be a good alternative immobilizing enzyme onto different support materials (membranes, microbeads, hollow fibers, nanoparticles, etc.). The immobilized enzymes are retained in a defined reaction space and can be reused continuously. Furthermore, immobilization has also been shown to increase enzyme stability [5]. Nanoparticles with unique physical, chemical, optical and electrical properties are frequently used in biotechnological applications. In addition, their ability to recognize biomolecules specifically has been the reason for their preference in biotechnological [6–9]. In addition, nanoparticles provide advantages such as minimum diffusion limitation, maximum surface area per unit mass, and high enzyme loading capacity [10]. It is possible to find various enzyme like catalase [11], α-amylase [12], laccase [13], inulinase [14], alcohol dehydrogenase [15], horseradish peroxidase and ester hydrolase [16], cellulase [17] immobilization studies in the literature.

Fumaric acid obtained from phthalic anhydride production. The application of fumaric acid in the industrial field is limited by its low solubility. In order to make it useful, it is enzymatically converted to L-malic acid, which is more soluble, and is used as an acidulant in fruit and vegetable juices, jams, infant foods, etc. [18,19]. Moreover, L-malate, which is used as an acidifier in the food and beverage industry, maintains 10% of its market share after citric acid. In addition, malic acid is also available in applications such as pharmaceuticals, electrode-less plating, metal coatings and paint infusions [3]. In all these processes, fumarase takes an enzymatic role in the production of malic acid. For these purposes, generally, microbial cells containing high amounts of enzyme are physically entrapped [20].

In the present work, the possibility of converting fumaric acid into L-malate, with fumarase bound on silanized nanostructures, is investigated. For this purpose, p(Hydroxyethyl methacrylate (p(HEMA)) nanostructures were synthesized by emulsion polymerization and silanized with 3-mercaptopropyltriethoxysilane (3-MTES) for immobilization of porcine heart fumarase enzyme. Covalent immobilization of the enzyme was predicted by the disulfide bond between the thiol groups on the enzyme and the thiol groups on the 3-MTES [21]. In the context of characterization of the synthesized nanoparticles, FTIR, SEM, zeta size analyzes were performed and the specific surface area of nanoparticles was calculated. The effect of initial enzyme concentration and pH on immobilization procedure were investigated and the change of Michaelis-Menten constants (K_m_ and V_max_) with immobilization was examined. The change in the storage stability of the enzyme by immobilization was also investigated.

## 2. Materıals and methods

### 2.1. Materials

Fumaric acid was provided by Pantochim (Belgium). Fumarase (EC 4.2.1.2) from porcine heart (F-1757) and L-malic acid were purchased from Sigma Co. (St. Louis, MO). All reaction and working solvents required in the experiments were taken from Merck Chemical Company (Darmstadt, Germany). The water used in the studies is distilled with the water destillation apparatus (ND 12 Nuve, Turkey).

### 2.2. Methods

#### 2.2.1. Synthesis of p(HEMA)-(3-MTES) nanostructures

**Figure 1:**
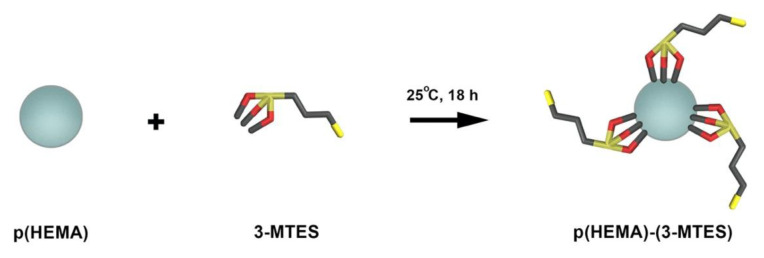
Silanization reaction of p(HEMA) nanoparticles by 3-MTES.

Firstly, we have synthesized p(Hydroxyethyl methacrylate) p(HEMA) nanostructures as described in our previous work with surfactant-free emulsion polymerization process [22], then we have silanized the synthesized p(HEMA) nanostructures by using 3-mercaptopropyltriethoxysilane (3-MTES). To summarize, we have solved 0.275 g of the stabilizer (PVA) in 25 mL of distilled water by heating. Then we have added the monomer, crosslinker and initiator to the PVA solution, respectively. We have transferred the mixture to a cylindrical reactor. In order to remove dissolved oxygen, we have treated the reactor with nitrogen gas for 1–2 min. Polymerization reaction carried out with 100 rpm in a shaking water bath at 70 °Cfor 24 h. After polymerization, we have washed the nanostructures repeatedly with methanol and distilled water to remove excess monomer and impurities. At the end of this washing procedure, resuspended nanostructures silanized by using 3-mercaptopropyltriethoxysilane (3-MTES). For this purpose, we have performed silanization procedure by mixing p(EGDM) nanostructures and 3-MTES (1:10, n/n) in ethanol (600 mL)-water (4 mL) mixture at 25 °Cfor 18 h. After silanization procedure, synthesized p(HEMA)-(3-MTES) nanostructures were washed several times. Finally, at the end of this washing procedure, resuspended nanostructures were stored in distilled water and stored at +4 °C(Figure 1).

#### 2.2.3. Characterization of p(HEMA)-(3-MTES) nanostructures

As part of the characterization study of nanostructures, we have taken the FTIR (Thermo Scientific Nicolet İS 50 FT-IR Spectrometer) spectrum of both p(HEMA) and p(HEMA)-(3-MTES) nanostructures to check the silanization process has taken place. For this purpose, we have created a pellet by mixing nanostructures (0.1 g) with KBr (0.1 g, IR Grade, Merck, Germany). Then we have taken the spectrum in the range of 4000–400 cm^−1^.

SEM photographs (Carl Zeiss 300 VP, Thermo Fisher Scientific FEI/Apreo S LoVac) of nanostructures were carried out to determine the surface morphology of nanostructures. Before SEM analysis, we have dried the nanostructures at 45 °C for 48 h and then powdered them.

We have analyzed with zeta sizer device (Malvern Instruments, Model 3000 HSA, England) to learn the dimensions of the nanostructures in liquid form and zeta potential values. Before analysis, we diluted the liquid nanostructures 50 times.

As the final stage of characterization studies, nanostructures’ surface area formula to calculate the specific surface area of p(HEMA)-(3-MTES) nanoparticles in m^2^/g [23]. The specific surface area of the p(HEMA)-(3-MTES) nanostructures were calculated by using following equation;


$$\text{N}=6\times 10^{10}\times \text{S}/\pi \times \rho \text{sx }{\text{d}}^{3}$$

In this equation, N is the number of nanostructures present in 1 mL suspension; ρs is the density of the bulk polymer (g/mL); S is % solid; d is the diameter (nm).

We have also calculated the value of the nanoparticles in 1 mL suspension in gram units by the mass-volume standard graph of the nanostructures.

#### 2.2.4. Production of L-Malate via immobilization of porcine heart fumarase on to p(HEMA)-(3-MTES) nanoparticles

**Figure 2:**
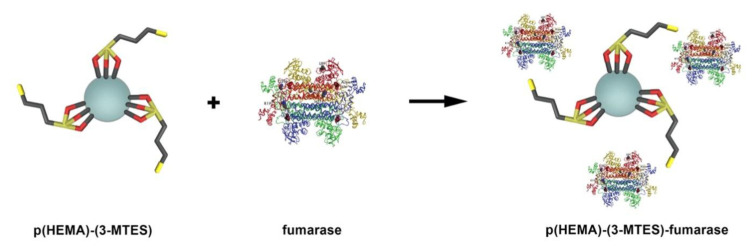
Schematic representation for immobilization of fumarase on p(HEMA)-3-MTES.

We have carried out the production of L-Malate via enzyme immobilization studies with a rotator (WiseMix RT-10, Visd) at room temperature under 120 rpm for 1 h in batch system. In order to investigate the effect of fumarase initial concentration on production of L-Malate via immobilization, we have prepared fumarase solutions in the range of 0.05 mM–0.25 mM by diluting the stock fumarase enzyme at different rates with 0.1 M pH 7.5 phosphate buffer. In pH studies, we have worked in the range of pH 4.5–8.5 (0.1 M acetate for 4.5, 5.5; 0.1 M phosphate for 6.5–8.5). We calculated the amount of adsorbed fumarase in U/g nanoparticle (Figure 2).

#### 2.2.5. Activity of free and immobilized fumarase for production of L-Malic acid

**Figure 3:**
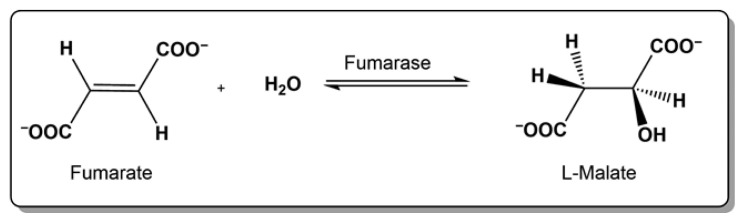
The reaction catalyzed by fumarase.

The enzyme apparently possesses an absolute specificity for the substrates fumarate and L-malate. At pH 7.5, the reaction on the right is faster than the one on the left, which is 100% at pH 9. The activity of fumarase is extremely sensitive to temperature and to the concentration and type of anion in the assay mixture. The molecular weight of the native enzyme is about 200,000 Da (Figure 3).

Activity measurements were performed at pH 7.5 in phosphate buffer (Na_2_HP0_4_/NaH_2_PO_4_) at concentrations between 0.01 and 0.5 M; sodium fumarate was then used as substrate by the enzyme. The reaction rate was determined by measuring the decrease in substrate concentration during this time. The fumaric acid concentration was measured spectrophotometrically at 310 nm. Calibration curves were prepared using standard fumaric acid concentrations in the range of 20–120 mM. This wavelength was selected by the spectra of fumarate and malate at between 400 and 200 nm. As reported by Colowick [24] and Giorno [5], fumarate shows good absorption in the ultraviolet region and at up to 320 nm, whereas malate has negligible absorption between the ultraviolet and visible range. To avoid interference with malate, we chose the 310-nm wavelength.

#### 2.2.6. Operational stability of immobilized fumarase for L-Malate production

In order to determine the operational stability of the free and immobilized enzyme for L-Malate production, free and immobilized fumarase were stored in 0.1 M pH:7.5 phosphate buffer for 30 days and their activities were measured periodically during this period.

## 3. Results and discussion

### 3.1. Characterization of p(HEMA)-3-MTES nanoparticles

**Figure 4:**
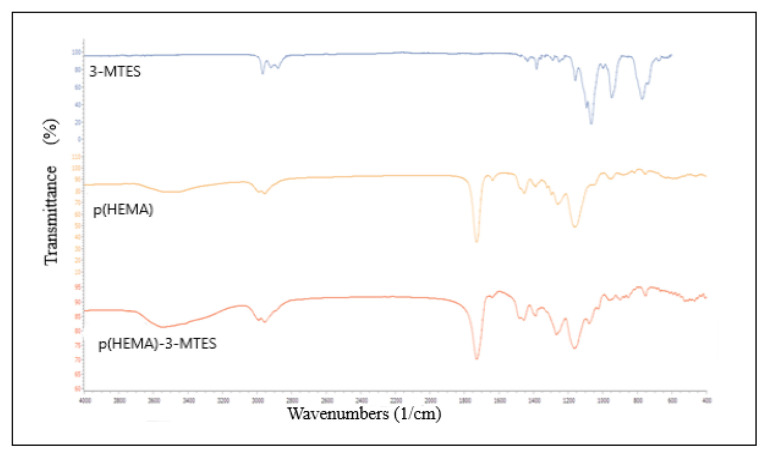
FTIR spectra of p(HEMA), p(HEMA)-3-MTES and 3-MTES.

In order to prove that p(HEMA) nanoparticle surfaces are silanized, FTIR spectra of both 3-MTES compound, p(HEMA) nanoparticles and p(HEMA)-3-MTES nanoparticles were taken. When the FTIR spectra of poly (HEMA) and p(HEMA)-3-MTES in Figure 4 are examined together, they belong to the stretching vibration of the broad band (O-H) observed around 3500 cm^−^1. Peaks observed around 3000–2886 cm^−1^ in all three spectra are the streching vibrations of alkyl groups. The band observed at about 1700 cm^−^ in the poly (HEMA) and poly (HEMA)-3-MTES spectrum belongs to stretching vibrations of n(C=O). The n(Si-O-C) and n(Si-O-R) groups are expected between 1200–1000 cm^−1^ in the IR spectrum. When the p (HEMA)-3-MTES spectra are examined in Figure 4, it can be said that the peak around 1100 cm^−1^ belongs to the n(Si-O-C) and n(Si-O-R) groups. Based on all the data, it is possible to say that 3-p(HEMA) nanoparticles and MTES reacted and the nanoparticle surface was successfully silanized (Figure 4).

**Figure 5a:**
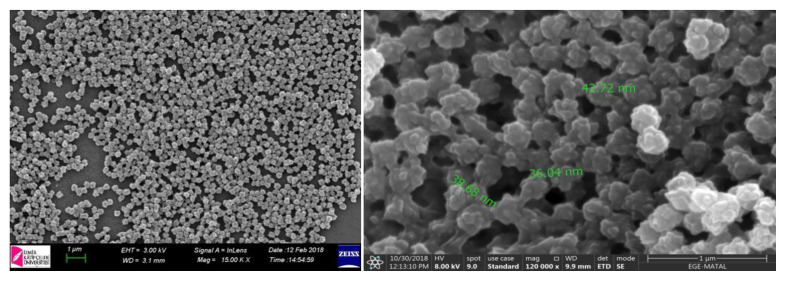
15,000 times magnified SEM images of p (HEMA)-3-MTES nanoparticles **b.** 120,000 times magnified SEM images of p(HEMA)-(3-MTES) nanoparticles.

SEM images were taken to make microscopic observations of the nanoparticles, to determine their morphological features, and determine their average size. Figures 5a and 5b show 15,000 and 120,000 times magnified images of nanoparticles, respectively. When Figure 5a is examined, it is seen that the average particle size is about 40 nm and it consists of homogeneously dispersed spherical particles, despite aggregation, which is a characteristic behavior of nanoparticles.

**Figure 6:**
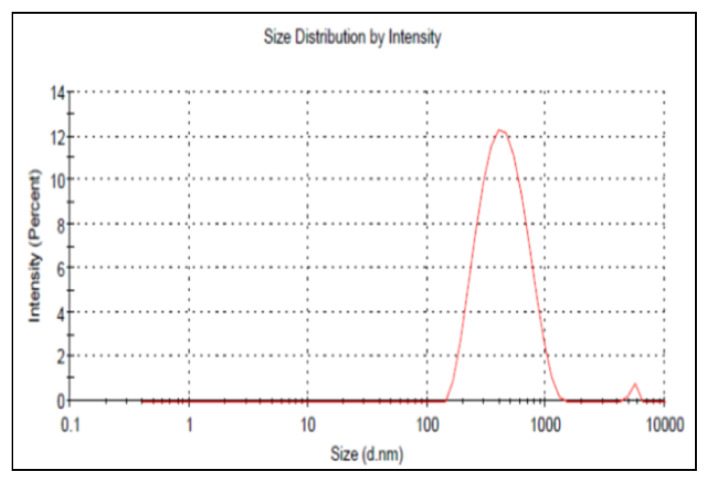
Zeta-size and size distribution of p(HEMA)-3-MTES nanoparticles.

The zeta potential value and size distribution of the nanoparticles were measured with a zetasizer. According to the zeta size results, the average size of the nanoparticles was 400 nm and the polydispersity was 0.22. The size mismatch between SEM measurements and zeta potential measurements can be explained as follows; SEM images were taken with dry and ground nanoparticles, and in zeta measurements, the particles were dispersed in distilled water. Since the nanoparticles could not be separated from each other completely in the zeta measurements, the sizes of the nanoparticles were measured large and the size distribution was spread over a wide range. All studies were carried out with nanoparticles dispersed in aqueous media and the surface area of nanoparticles was calculated according to the zeta size results. According to these calculations, the surface area of the nanoparticles was found to be 461.11 m^2^/g polymer (Figure 6).

### 3.2. Production of L-Malate via immobilization of fumarase on to p(HEMA)-3-MTES nanoparticles for L-Malate production

**Figure 7:**
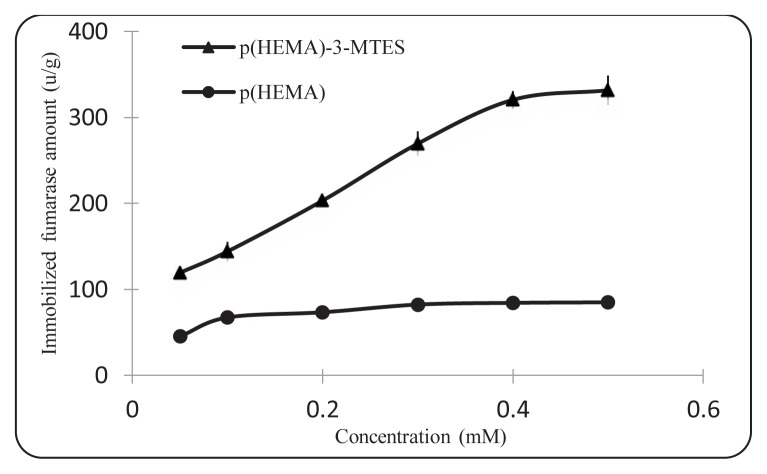
Effect of initial concentration on immobilization (0.1 M pH: 7.5 phosphate buffer, 25 ^o^C).

The effect of initial enzyme concentration on immobilization for L-Malate production was investigated. As can be seen in Figure 7, the amount of immobilized enzyme increases as the fumarase concentration increases for L-Malate production. A plateau value (320.33 u/g) was reached for the immobilized enzyme at 0.4 mM fumarase concentration. It was also observed that the amount of nonspecific fumarase bound to the unsilanized p(HEMA) nanoparticles was significantly less than the amount of immobilized fumarase in the surface silanized p(HEMA)-3-MTES nanoparticles.

**Figure 8:**
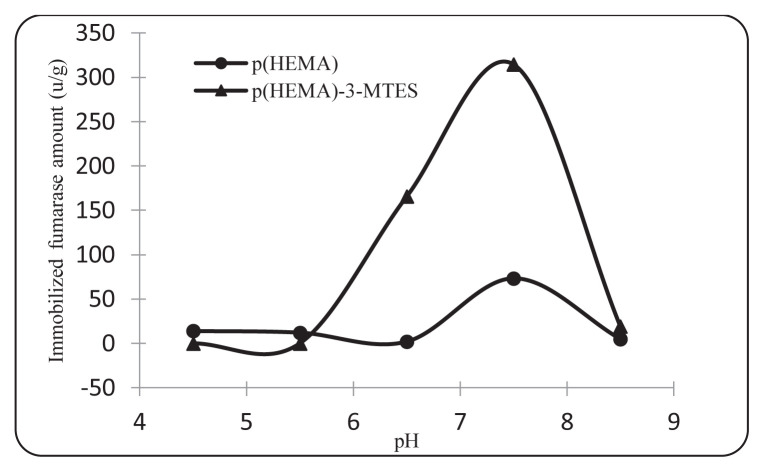
Effect of pH on immobilization (0.4 mM fumarase concentration, 25 ^o^C.

To examine the effect of medium pH on immobilization for L-Malate production and to determine the optimum pH for fumarase immobilization, pH 4.5–8.0 (0.1 M acetate for 4.5, 0.1 M phosphate for 5.5, 0.1 M phosphate for 6.5–8.5) was studied.. As shown in Figure 8, the maximum immobilization capacity was observed for p(HEMA)-3-MTES nanoparticles at pH 7.5 (314.5 u/g). The amount of fumarase immobilized on the p (HEMA) nanoparticles at pH 7.5 was 73.3 u/g.

### 3.3. Activity studies

**Figure 9:**
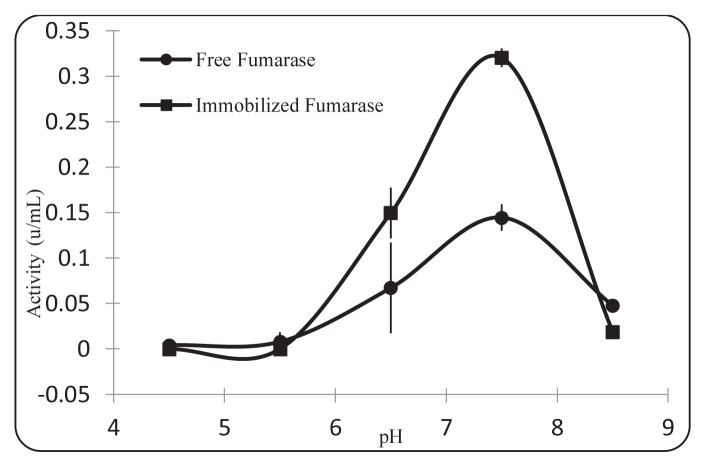
Effect of pH on activity of free fumarase and immobilized fumaraze (0.4 mM fumarase concentration, 25 ^o^C).

The effect of pH on fumarase activity was investigated after fumarase immobilization to silanized nanostructures for L-Malate production. As seen in Figure 9, the optimum pH value of the enzyme did not change after the fumarase was immobilized in the silanized nanoparticles. The optimum pH value for both free fumarase and immobilized fumarase was observed as pH 7.5.

Michaelis-Menten constants (Km and Vmax values) were calculated for free and immobilized fumarase for L-Malate production. As seen in Table, the Km values of free and immobilized fumarase are 5.55 mM and 5.05 mM, respectively. The Km value indicates the affinity of an enzyme for its substrate. As the Km value increases, the affinity of the enzyme to its substrate decreases [25]. Since the Km value of the immobilized enzyme is lower than the Km value of the free enzyme, it can be said that the affinity of fumarase with its substrate increases with immobilization. The Vmax value indicates the maximum speed reached when the enzyme is saturated with the substrate [12]. In our study, an increase in fumarase rate was observed after immobilization.

**Figure 10:**
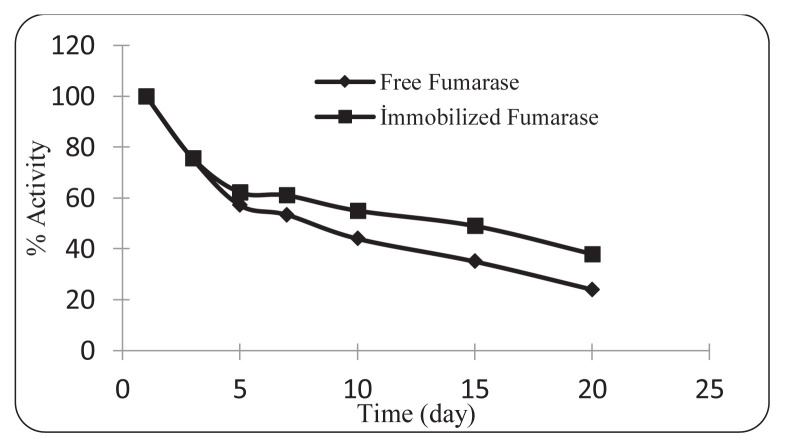
Storage stability of free fumarase and immobilized fumarase (0.4 mM fumarase concentration, 0.1 M pH: 7.5 phosphate buffer, 25 ^o^C).

The effect of immobilization on the operational activity of fumarase in L-Malate production was investigated. For this, free and immobilized fumarase solutions were stored in 0.1 M pH 7.5 phosphate buffer and their activities were regularly measured for 30 days. Figure 10 shows the change in the operational activity of free and immobilized fumarase over 30 days. At the end of 30 days, the free enzyme retained 10% of its initial activity, while the immobilized enzyme retained 25% of its initial activity. This shows that the operational activity of fumarase immobilized nanopreparations developed for L-Malate production is approximately 2.5 times higher than that of free enzyme preparations.

## 4. Conclusion

Fumarase enzyme, which is involved in the Krebs cycle in metabolism, is of great importance in medicine and industry. For L-malate production, it is possible to use this enzyme more economically, more stable and more functionally by immobilization on carrier supports without losing its activity. On the other hand, having a large surface area and lack of internal diffusion resistance make nanomaterials good carriers [23]. In this study; the enzymatic production of L-malate is carried out using a batch system with fumarase immobilized silanized nanoparticles. Nanopreparations were prepared by immobilizing fumarase enzyme on p(HEMA)-3-MTES nanoparticles. The usability of these silanized nanopreparations in L-Malate production was investigated. In the study, when the activities of the nanopreparations developed for use in the production of L-Malate were compared with the free fumarase activity, it was observed that there was a significant increase in the activities of the nanopreparations. In addition, it was determined that this activity during L-Malate production would be maintained 2.5 times better than free fumarase for 30 days. With the results obtained in this study, it is possible to say that these fumarase immobilized nanopreparations, which are prepared for use in L-Malate production, can be used in L-Malate production with higher activity and lower cost compared to free fumarase. As a result, the synergistic effect of catalysis and separation was maximized in this study, in which the usability of the developed fumarase-immobilized nanopreparations in L-Malate production was investigated. With the developed strategy, it will be possible to produce L-Malate with lower cost and higher efficiency. These nanopreparations developed for use in L-Malate production should be tested by optimizing the production conditions in a pilot facility to be developed. With subsequent scale-up studies, it is possible to use them in L-Malate production with low cost and high efficiency.

## Figures and Tables

**Table:** Michaelis-Menten constants of immobilized and free fumarase.

	K_m_ (mM)	V_max_ (u/mL)	R^2^
**Free fumarase**	5.55	0.26	0.989
**Immobilized fumarase**	5.05	0.466	0.994
